# Response and Adverse Effects to Lithium Treatment in Patients With Bipolar Disorder at Amanuel Mental Specialized Hospital, Addis Ababa, Ethiopia

**DOI:** 10.1155/bmri/8884457

**Published:** 2026-03-17

**Authors:** Abebe Ejigu Hailu, Solomon Teferra, Ephrem Engidawork

**Affiliations:** ^1^ Department of Pharmacology and Clinical Pharmacy, School of Pharmacy, Addis Ababa University, Addis Ababa, Ethiopia, aau.edu.et; ^2^ Department of Psychiatry, School of Medicine, Addis Ababa University, Addis Ababa, Ethiopia, aau.edu.et

**Keywords:** adverse effects, bipolar disorder, lithium, mood stabilizers, treatment response

## Abstract

This study assessed the clinical response and adverse effects of lithium in patients with bipolar disorder (BD) at Amanuel Mental Specialized Hospital (AMSH) in Addis Ababa, Ethiopia. A retrospective cohort and cross‐sectional design were employed as part of the Neuropsychiatric Genetics of African Populations (NeuroGAP)‐Psychosis, Ethiopia project including 262 patients on lithium therapy for at least 6 months; adverse effects were evaluated in 157 of them. Clinical response was measured using the Alda scale, and adverse effects were assessed via a structured questionnaire. Among the participants, 27.5% were classified as good responders (GRs), whereas the remaining 72.5% were classified as insufficient responders (IRs), comprising both partial and nonresponders. Significant differences were observed in age group, length of lithium therapy, concomitant therapy, and number of concomitant medications, with 33% of the IRs on lithium for 1–2 years compared to only 10% of the GRs; most patients in both groups had been on lithium for 2–5 years. Additionally, 83.2% of the IRs used other medications alongside lithium, with 78% using just one additional medication. Logistic regression found that age (*p* = 0.008), concurrent use of other psychiatric medications (*p* < 0.001), and stable lithium plasma levels (*p* = 0.003) were significantly associated with lithium response. Regarding adverse effects, 58.6% of patients reported at least one adverse event, with tremors, excessive thirst, and frequent urination being the most common. Age, tobacco use, and treatment duration were associated with lithium adverse effects, with younger age and tobacco use linked to higher risk, whereas older age and longer treatment were associated with fewer adverse effects. In conclusion, the findings highlight the importance of monitoring use of psychiatric concomitant medications and plasma lithium levels to improve outcomes in BD patients on lithium.

## 1. Introduction

Bipolar disorder (BD) is a mood condition characterized by severe episodes of mania and depression. Although it is often referred to as a “cyclic” illness, one defining feature is the presence of manic episodes though these symptoms can also be present in other conditions [1]. BD is linked to extremely high rates of morbidity and mortality. A study conducted by Perlis et al. (2010) [2] suggested that the disorder carries a 20‐fold increased risk of suicide when compared to the general population. The condition can be severely crippling, leading to a significant financial burden, and there are significant personal and societal costs associated with BD. Relapse is also prevalent among those receiving appropriate treatment [3].

A small percentage of the limited resources available to the health system as a whole is designated for mental health treatment in developing countries, and BD consumes a substantial portion of this mental health resource. This is precisely the situation in Ethiopia, where mental health services are the least developed, health facilities are severely understaffed, and budgetary deficits are a problem [4]. Previous studies in Ethiopia have consistently demonstrated that mental health issues are common problems in the country [5–8]. Notably, BD has higher reported lifetime prevalence in men (0.6%) compared to women (0.3%) [9].

Based on how the disease progresses, BD treatment is determined by whether a patient is experiencing manic/hypomanic, episode with mixed features, or depressed episodes [10]. The most significant pharmacological therapy for reducing manic symptoms and depression are mood stabilizers like lithium and lamotrigine, respectively. Since lithium effectively treats and prevents both the manic and depressed phases of BD albeit with a stronger antimanic effect than an antidepressant one, it is also regarded as a model mood stabilizer. In fact, lithium is still advised as a first‐line treatment for patients with BD despite the growing availability of new psychoactive medications and shifting diagnostic standards for mental illnesses [11, 12].

Individual responses to treatment can vary greatly, and some patients with BD show only partial or no response at all. Although lithium is effective for most people, 30%–40% do not respond to it. Additionally, many patients cannot continue lithium therapy due to intolerable adverse effects, such as kidney damage, weight gain, acne, thyroid suppression, and other complications [13, 14]. Despite its well‐known and varied effectiveness, lithium has a particularly problematic profile when it comes to clinical adverse effects, and its therapeutic index is very narrow. Although various guidelines exist for the pharmacological treatment of BDs, there has been limited research on long‐term medication response and its clinical correlates in patients with BD receiving routine care. This study is aimed at assessing the clinical response and adverse effects of long‐term treatment with lithium in individuals with BD.

## 2. Methodology

### 2.1. Study Area and Patients

The study was conducted at Amanuel Mental Specialized Hospital (AMSH), Addis Ababa, Ethiopia, which is the only mental health hospital in the country. AMSH is one of the oldest hospitals established by the Italians in 1938. It is located in Addis Ketema Subcity, western part of Addis Ababa. The hospital has a bed capacity of 300, with 250 medical specialists, having varying levels of mental health training and expertise, and 344 support staff members. On average, 400 ambulatory patients receive medical and counseling services daily.

This study is a retrospective cohort and cross‐sectional analysis. A retrospective cohort design was used to assess lithium′s response through chart review to determine clinical outcomes over time, whereas a cross‐sectional design was applied for the assessment of adverse effects based on patient interviews capturing cumulative adverse effects experienced during treatment. This is part of the Neuropsychiatric Genetics of African Populations‐Psychosis, Ethiopia (NeuroGAP‐P‐E) project. This project is conducted across four countries in eastern and southern Africa: Ethiopia, Kenya, Uganda, and South Africa, with data collection occurring at five different sites. In Ethiopia, data are specifically collected at AMSH. Eligible participants for NeuroGAP included individuals who either have a clinical diagnosis of BD or schizophrenia, or those without any history of psychosis [15]. For this specific study, a more selective recruitment process was employed for patients diagnosed with BD as part of the broader NeuroGAP initiative. Participants diagnosed with BD and on lithium therapy for at least 6 months were eligible for this study. All participants were at least 18 years old and provide written informed consent or a fingerprint in case of illiteracy. To ensure that participants have the capacity and autonomy to consent, the University of California, San Diego Brief Assessment of Capacity to Consent (UBACC) was used. Individuals were excluded if they lack a diagnosis of BD, exhibit severe and intrusive psychiatric symptoms at the time of consent, are intoxicated or in withdrawal from alcohol or substance abuse, are under involuntary detention, lack fluency in a language the consent form has been translated into, or are determined by the UBACC to lack the capacity to consent. We obtained 614 patients with BD from the NeuroGAP project (April 2018–January 2021) and reviewed their medical records. From this cohort, a more selective recruitment process identified 262 patients with BD who had been on lithium treatment for at least 6 months. However, we were able to contact only 157 of them for the adverse effect interview.

### 2.2. Data Collection and Management

Chart review and interview were used to collect relevant clinical and sociodemographic information. Chart review was conducted by the research staff of the NeuroGAP project, including a psychiatrist who received specific training on extracting clinical data from medical records using a standardized tool. Patient interviews were performed by trained physicians to confirm diagnoses and gather information on sociodemographic and clinical characteristics. Demographic information included age, gender, education, marital status, among others. Clinical data included age at which mood episodes start and how frequently they do so, the presence and frequency of psychotic symptoms, hospitalization, use of alcohol, tobacco, and other drugs, and a family history of mental illness. Data quality was maintained by using standardized data collection forms, training of data collectors, verifying participant information with the source document, and periodic quality audit. Evaluation was conducted using the following instruments:

• Mini International Neuropsychiatric Interview (MINI) Version 7.0.2 for DSM‐5: includes modules A, C, K, and O, covering major depressive episodes, manic and hypomanic episodes, psychotic disorders, and mood disorders with psychotic features, respectively [16].
• Life Events Checklist for DSM‐5: a 17‐item scale assessing exposure to potentially traumatic events [17].
• Alcohol, Smoking, and Substance Involvement Screening Test (ASSIST) V3.0: focuses on substance type and usage both over the participant′s lifetime and within the past 3 months [18].
• Composite International Diagnostic Interview (CIDI) Screener: a checklist within the CIDI covering chronic physical conditions, including diabetes, HIV/AIDS, epilepsy/seizures, and tuberculosis [19].

### 2.3. Lithium Response Measurement

The lithium response phenotype was evaluated using the Retrospective Criteria of Long‐Term Treatment Response in BD research subjects, operationalized through structured retrospective assessment. The clinical course was compared between periods without lithium treatment and during adequate lithium treatment. The off‐treatment period was defined as the interval from the onset of BD to the initiation of continuous lithium therapy, while the on‐treatment period was defined as the time from lithium initiation to the most recent clinical follow‐up, with a minimum lithium exposure of at least 6 months. Assessment was based on retrospective review of clinical records, including outpatient psychiatric clinic notes, hospitalization and discharge summaries, and documented medication histories. Clinical evaluation focused on differences in the frequency, duration, and severity of mood episodes, hospitalization rates, and the need for additional mood‐stabilizing interventions between the two periods. The plasma lithium level, adherence to lithium treatment, and other relevant clinical data were also extracted from the chart. Plasma lithium levels were defined using steady‐state concentrations measured ≥ 5 days after any dose change, with at least two values recorded ≥ 3 months apart. Levels were classified as stable if all measurements were within the therapeutic range (0.6–1.2 mEq/L) with low variability (coefficient of variation ≤ 20%), and fluctuating if any value was outside range or variability exceeded 20%. When retrospective data were incomplete or ambiguous, all available longitudinal data were carefully integrated, and response was assessed conservatively based on the most reliable documented evidence.

### 2.4. Alda Score

The degree of lithium response was quantified using the Alda scale, which comprises two main components: Criterion A and Criterion B. Criterion A measures the extent of clinical improvement during lithium treatment compared with the prelithium period and is scored from 0 (*no improvement or worsening*) to 10 (*complete remission*). In this study, Criterion A was operationalized in accordance with the original Alda methodology and subsequent validation studies [20]. Criterion A scoring involved comparison of episode frequency, severity, hospitalization rates, and the need for additional mood‐stabilizing interventions between the off‐treatment and on‐lithium periods, using standard Alda anchor points. Criterion A scoring relied on retrospective data described above.

Criterion B assesses the causal link between treatment and improvement, with subscores B1 through B5. B1 and B2 rate episode number and frequency (higher scores mean fewer episodes), B3 rates lithium treatment duration (higher scores mean shorter treatment), B4 rates adherence (higher scores reflect worse adherence), and B5 rates additional psychotropic use during stable periods (higher scores indicate higher use and duration) [20, 21].

The total Alda score is calculated by subtracting the sum of the B scores from the A score. To determine a causal link between lithium treatment and clinical improvement, it is crucial to compare the clinical course (number, frequency, and severity of episodes) during lithium treatment versus nontreatment periods, along with ensuring good drug adherence and minimal impact from other medications (e.g., hypnotics, antidepressants, antipsychotics, and mood stabilizers). Studies suggest that the optimal cutoff for distinguishing nonresponders (NRs) (Scores 0–6) from responders (Scores 7–10) to lithium is a total score of 6 or 7 [22, 23]. According to the available literature [20, 24] patients with a total score of ≥ 7 were characterized as good responders (GRs), patients with a total score of ≤ 3 were characterized as NRs, and the remaining individuals were classified as partial responders (PaRs), which collectively with the NRs form the insufficient responders (IRs) group. Retrospective Alda scoring was conducted by a single research psychiatrist with formal clinical training in psychiatry and experience in BD and lithium pharmacotherapy, following standardized training on the Alda scale. Scoring was based on structured review of outpatient and inpatient records using standardized scoring guidance, predefined operational definition, and structured abstraction form with conservative handling of incomplete or ambiguous clinical histories. External validation was performed on a subset of patients for whom clinician‐documented treatment response was available, supported by hospitalization patterns, showing consistent agreement. Additionally, a sensitivity analysis excluding cases with ambiguous or incomplete documentation was conducted, with results remaining materially unchanged.

### 2.5. Lithium Adverse Effects

The assessment of adverse effects was conducted with a questionnaire similar to that used by Ghose (1977) [25], translated into Amharic and subsequently back‐translated into English. All 20 adverse effects were evaluated and ranked using a three‐point grading system, with zero representing no adverse effects, one representing mild adverse effects, two representing moderate adverse effects, and three representing severe adverse effects. The questionnaire also enquires about the patient′s adherence to medication, lithium dosage, duration of treatment, and the type and quantity of other psychotropics supplied to them while on lithium treatment. Finally, patients were asked if their doctors had ever advised them to discontinue taking lithium, and if so, why. The adverse effect questionnaire was completed by either a mental health nurse or a project researcher using the Amharic language version.

### 2.6. Ethical Consideration

This study is part of the NeuroGAP project, which received ethical approval from the Institutional Review Board of the College of Health Sciences, Addis Ababa University (protocol 014/17/Psy) and also obtained specific approval for this subproject from the same IRB (protocol 085/18/Pharmacy). Permission to conduct the study was also obtained from AMSH. Furthermore, for the interview component, the subjects or their legal guardians provided written informed consent after being informed about the study′s objectives, procedures, anticipated results, potential hazards, and associated discomforts. The UBACC score was used during consent to ensure that participants understand the study, their requirements, and withdrawal rights. Those who passed the score provided written consent. For the retrospective chart review, data were extracted from medical records, and patient informed consent was waived. Additionally, confidentiality was ensured by using an ID system that prevents linking participants to their data.

### 2.7. Statistical Analysis

All analyses were performed using SPSS software package, Version 26. The data were sorted and coded for SPSS, and descriptive statistics were used to present sociodemographic, anthropometric, clinical, diseases course characteristics, medication use, and adverse effects. Continuous variables are presented as mean ± SEM and categorical variables as percent proportions. Nonparametric descriptive statistical tests (*χ* ^2^) were used to compare GR and IR groups. When analyzing categorical variables with multiple subcategories, we applied post hoc pairwise comparisons with Bonferroni adjustments where relevant to control for Type I errors. Each sociodemographic, anthropometric, and clinical characteristic was first evaluated using univariate logistic regression to assess its association with treatment response and adverse effects. Candidate variables were defined a priori based on clinical relevance and evidence from the literature and were additionally screened using bivariate analyses; variables with *p* < 0.20 in univariate analysis or with strong clinical plausibility were included in the multivariable logistic regression models. Backward‐stepwise logistic regression was used in the multivariable analysis to identify independent predictors of the primary outcome. We checked for multicollinearity prior to conducting the analysis using standard diagnostic methods (such as variance inflation factor, VIF < 5) to ensure that it did not affect interpretability and predictive capacity of the model. A statistically significant correlation between the independent and dependent variables was declared at *p* < 0.05.

## 3. Results

We assessed the response of 262 BD patients on lithium for at least 6 months. Information on adverse effects, however, was available only for 157 (59.9%) as these were collected through interview with patients who could be traced. The data for lithium response and adverse effects were collected independently. Lithium response data were obtained retrospectively for all patients from medical records, independent of the adverse effect assessment. Baseline characteristics were compared between contacted and noncontactable patients, and no meaningful differences were observed.

### 3.1. Sociodemographic and Clinical Characteristics of the Study Participants

There was a female gender preponderance in both the response and adverse effect category, with the ratio being 56.1% versus 43.9% and 58% versus 42%, respectively (Table 1). Over half never married, and the majority still live with their parents in both categories. The majority of patients in the lithium response group attended secondary school (22.4%), whereas the lithium adverse effects group (22.3%) had completed college. Furthermore, the majority of patients were from Oromo and Amhara ethnic groups. Substance use patterns indicated 27.0% were tobacco users, 43.3% consumed alcohol, and 34.2% used khat, whereas cannabis use was minimal. Among the individuals evaluated, 80.6% had no hospital admissions, while 18.6% had at least one admission in the response group. The corresponding figures for the adverse effect group were 82.2% and 17.8%, respectively. The most common comorbid conditions included severe headache (13.0%), chronic back problems (11.5%), and arthritis (8.4%). Other notable conditions were hypertension (7.3%), seasonal allergies (5.0%), tuberculosis (4.6%), ulcers (4.6%), HIV (4.2%), and diabetes (4.2%).

**Table 1:** Descriptive sociodemographic and clinical characteristics of lithium‐treated patients at AMSH, Ethiopia (April 2018–January 2021).

Variables	Categories	Response study (%) (*n* = 262)	Adverse effect study (%) (*n* = 157)
Sex	Male	115 (43.9)	66 (42.0)
	Female	147 (56.1)	91 (58.0)
Age group	18–29.9	78 (29.8)	49 (31.2)
	30–44.9	137 (52.3)	77 (49.1)
	≥ 45	47 (17.9)	31 (19.7)
Marital status	Never married	139 (53.1)	87 (55.4)
	Married	74 (28.2)	41 (26.1)
	Divorced	30 (11.4)	18 (11.5)
	Widowed	11 (4.2)	6 (3.8)
	Separated	8 (3.1)	5 (3.2)
Current living arrangement	Lives alone	46 (17.6)	27 (17.2)
	Lives with spouse/partner	73 (27.9)	40 (25.5)
	Lives with parents	98 (37.4)	60 (38.2)
	Lives with relatives	41 (15.4)	28 (17.8)
	Others	3 (1.1)	1 (0.6)
Level of education	No education	8 (3.1)	5 (3.2)
	Did not complete primary school	52 (19.9)	30 (19.1)
	Completed primary school	15 (5.7)	11 (7.0)
	Did not complete secondary school	59 (22.4)	30 (19.1)
	Completed secondary school	44 (16.7)	28 (17.8)
	Did not complete college education	29 (11.0)	17 (10.8)
	Completed college education	54 (20.5)	35 (22.3)
Ethnicity	Oromo	82 (31.2)	48 (30.6)
	Amhara	89 (33.5)	53 (33.8)
	Sebat bet Gurage	38 (14.4)	27 (17.2)
	Sodo Gurage	15 (5.7)	6 (3.8)
	Gedeo	2 (0.8)	—
	Shaakicho	1 (0.4)	1 (0.6)
	Silte	13 (4.9)	7 (4.5)
	Tigrie	9 (2.3)	4 (2.5)
	Hadiyya	6	5 (3.2)
	Wolane	2 (0.8)	1 (0.6)
	Wolayitta	2 (0.8)	2 (1.3)
	Others	3 (1.20)	2 (1.3)
Tobacco use	Yes	71 (27.0)	42 (26.8)
	No	191 (72.0)	115 (73.2)
Alcohol use	Yes	114 (43.3)	69 (43.9)
	No	148 (56.5)	88 (56.1)
Khat use	Yes	90 (34.2)	56 (35.7)
	No	172 (65.4)	101 (64.3)
Cannabis use	Yes	6 (2.3)	3 (1.9)
	No	256 (97.3)	154 (98.1)
Body mass index	< 18.5	4 (1.5)	3 (1.9)
	18.5–24.9	127 (48.3)	73 (46.5)
	25–29.9	76 (28.9)	50 (31.8)
	≥ 30	55 (20.9)	31 (19.7)
Hospital admission	No	212 (18.6)	129 (82.2)
	Yes	49 (80.6)	28 (17.8)
Comorbid conditions	Arthritis	22 (8.4)	15 (9.6)
	Chronic back problem	30 (11.5)	17 (10.8)
	Severe headache	34 (13.0)	20 (12.7)
	Other chronic pain	4 (1.5)	2 (1.3)
	Seasonal allergies	13 (5.0)	6 (3.8)
	Stroke	1 (0.4)	0
	Heart attack	1 (0.4)	1 (0.6)
	Heart diseases	2 (0.8)	2 (1.3)
	Hypertension	19 (7.3)	14 (8.9)
	Asthma	5 (1.9)	2 (1.3)
	Tuberculosis	12 (4.6)	8 (5.1)
	Chronic obstructive pulmonary disease	6 (2.3)	4 (2.5)
	Diabetes mellitus	11 (4.2)	6 (3.8)
	Ulcer	12 (4.6)	5 (3.2)
	HIV	11 (4.2)	8 (5.1)
	Epilepsy	2 (0.8)	1 (0.6)
	Other conditions	—	4 (2.5)

### 3.2. Lithium Response

The mean Alda A score was 8.7 (SD = 1.07), and the mean total score was 5.18 (SD = 1.803) (Table 2). According to the Alda score, 72 (27.5%) participants were GRs, whereas the rest 190 (72.5%) were IRs, including 69.4% of PaRs and 3.1% of NRs.

**Table 2:** Total and individual scores for the ALDA scale.

S/N		*N*	Mean	SEM	Median	Mode	SD
1	ALDA A	262	8.7	0.066	9	9	1.07
2	ALDA B1	262	0.96	0.035	1	1	0.562
3	ALDA B2	262	0.99	0.03	1	1	0.479
4	ALDA B3	262	0.53	0.044	0.00	0	0.715
5	ALDA B4	262	0.24	0.029	0.00	0	0.471
6	ALDA B5	262	0.81	0.030	1.00	1	0.489
7	ALDA BT	262	3.52	0.085	3	3	1.372
8	Total (A–B)	262	5.18	0.111	5.00	7	1.803

#### 3.2.1. Comparison of Demographic and Disease Course Characteristics

The clinical history and demographic characteristics of both IRs and GRs are shown in Table 3. In terms of gender, marital status, type of housing, and educational attainment, there were no notable differences between the two groups. There were also no discernible differences between the two groups in the occurrence of other conditions observed in the current subjects, such as alcohol use, smoking status, cannabis use, body mass index (BMI), comorbid conditions, hospital admission, lithium dosage, use of nonpsychiatric medications, being a psychiatric inpatient, and level of adherence. However, age group (*p* = 0.012), length of lithium therapy (*p* < 0.001), concomitant therapy (*p* < 0.001), and number of concomitant medications (*p* = 0.001) showed a significant difference. Around 33% of the IRs took lithium for 1 up to 2 years, but only 10% of the GRs were in this category. Most of the patients in both groups took lithium from 2 up to 5 years (70.8% of GRs and 40.5% of the IRs). In addition, most of the IRs (83.2%) used additional medications with lithium, of which 78% of them used additional one medication.

**Table 3:** Baseline clinical and sociodemographic characteristics of good and insufficient lithium responders among patients with bipolar disorder at AMSH, Ethiopia (April 2018–January 2021).

Sociodemographic		Good responders (%) (*n* = 72)	Insufficient responders (%) (*n* = 190)	*χ* ^2^/*t*‐test	*p* value
Sex	Female	43 (59.7)	104 (54.7)	0.527	0.468
	Male	29 (40.3)	86 (45.3)		
Age	Mean (SD)	37.07 (8.3)	34.94 (9.7)	−1.646	0.101
Age category	18–29.9	13 (18.0)	65 (34.2)	8.894	0.012 ^∗^
	30–44.9	48 (66.7)	89 (46.8)		
	≥ 45	11 (15.3)	36 (19.0)		
Marital status	Never married	35 (48.6)	104 (54.7)	1.406	0.843
	Married	22 (30.6)	52 (27.4)		
	Divorced	8 (11.1)	22 (11.6)		
	Widowed	4 (5.5)	7 (3.7)		
	Separated	3 (4.2)	5 (2.6)		
Current living arrangement	Lives alone	15 (20.8)	31 (16.3)	4.705	0.319
	Lives with spouse/partner	22 (30.6)	51 (26.8)		
	Lives with parents	21 (29.2)	77 (40.5)		
	Lives with other relatives	14 (19.4)	27 (14.2)		
	Others	0 (0.0)	3 (1.6)		
Level of education	No education	4 (5.6)	4 (2.1)	11.717	0.069
	Did not complete primary school	8 (11.1)	44 (23.2)		
	Completed primary school	3 (4.2)	12 (6.3)		
	Did not complete secondary school	24 (33.3)	35 (18.4)		
	Completed secondary school	12 (16.7)	32 (16.8)		
	Did not complete college education	8 (11.1)	21 (11.1)		
	Completed college education	13 (18.1)	41 (21.7)		
Tobacco use	Yes	22 (30.6)	49 (25.8)	0.600	0.438
	No	50 (69.4)	141 (74.2)		
Alcohol use	Yes	37 (51.4)	77 (40.5)	2.507	0.113
	No	35 (48.6)	113 (59.5)		
Khat use	Yes	28 (38.9)	62 (32.6)	0.907	0.341
	No	44 (61.1)	128 (67.4)		
Cannabis use	Yes	2 (2.8)	4 (2.1)	0.106	0.745
	No	70 (97.2)	186 (97.9)		
Body mass index	< 18.5	1 (1.4)	3 (1.6)	0.909	0.823
	18.5–24.9	33 (45.8)	94 (49.5)		
	25–29.9	24 (33.3)	52 (27.4)		
	≥ 30	14 (19.4)	41 (21.6)		
Hospital admission	No	61 (84.7)	151 (79.9)	0.797	0.372
	Yes	11 (15.3)	38 (20.1)		
Comorbid conditions	No	37 (51.4)	108 (56.8)	0.628	0.428
	Yes	35 (48.6)	82 (43.2)		
Currently psychiatric inpatient		4 (5.6%)	20 (10.5)	1.529	0.216
Presence of other psychiatric diagnosis		21 (29.2)	46 (24.2)	0.674	0.412
Current no. of psychiatric medications	Only one medication	14 (19.4)	36 (18.9)	0.15	0.928
	Two medications	51 (70.8)	135 (71.0)		
	Three medications	6 (8.3)	19 (10)		
Nonpsychiatric medications		8 (11.1)	16 (8.4)	0.454	0.500
Level of Li adherence	Not available	6 (8.3)	18 (9.5)	1.31	0.727
	No adherence problem	56 (77.8)	135 (71.1)		
	Intermediate adherence/some treatment interruption	9 (12.5)	33 (17.4)		
	Poor adherence/greater treatment interruption	1 (1.4)	4 (2.1)		
Li dose category	300 mg	3 (4.2)	5 (2.6)	3.681	0.298
	600 mg	18 (25)	44 (23.2)		
	900 mg	47 (65.3)	115 (60.5)		
	1200 mg and above	4 (5.6)	26 (13.7)		
Duration of Li therapy	Less than 1 year	7 (9.7)	43 (22.6)	27.552	*p* < 0.001^∗^
	1–2 years	7 (9.7)	62 (32.6)		
	2–5 years	51 (70.8)	77 (40.5)		
	> 5 years	7 (9.7)	8 (4.2)		
Concurrent medications with Li	No	28 (38.9)	32 (16.8)	14.374	*p* < 0.001^∗^
	Yes	44 (61.1)	158 (83.2)		
No. of concurrent medications with Li	No other medication	28 (38.9)	32 (16.8)	16.617	0.001 ^∗^
	One medication	44 (61.1)	149 (78.4)		
	Two medications	0 (0.0)	7 (3.7)		
	Three medication	0 (0.0)	2 (1.1)		

*Note:* Nonpsychiatric medications refers to the last recorded medications used during data collection time.

Abbreviation: SD, standard deviation.

^∗^ *p* < 0.05.

#### 3.2.2. Predictors for Lithium Response

The univariate analysis included sex, age category, tobacco use, khat use, alcohol use, hospital admission status and number of hospital admissions, presence of comorbidities, BMI category, psychiatric inpatient status, presence of other psychiatric diagnoses, level of adherence, lithium dose, duration of lithium therapy, plasma lithium level, use of concurrent medications with lithium, and number of concurrent medications. This analysis has shown that age group, alcohol consumption, concurrent use of other psychiatric drugs, blood lithium level, and dose of lithium had a relationship with response (Table 4). Thus, these variables were included in the multivariable binary logistic regressions. The Hosmer–Lemeshow test did not indicate poor model fit (*χ* ^2^ = 8.01, df = 8, *p* = 0.433); model discrimination was also supported by the area under the ROC curve (C‐statistic), indicating acceptable predictive performance. Three factors (age category, lithium plasma level, and concurrent use of other psychiatric medications) were shown to be strongly correlated with lithium response in the multivariable analysis. Accordingly, individuals with age category from 30 to 44.9 were more likely to respond to lithium treatment compared with those with age 18–29.9 years (AOR = 2.86; 95% CI: 1.31–6.23, *p* = 0.008). On the other hand, those who use concurrent medications along with lithium had lower odds of responding to lithium treatment (AOR = 0.253; 95% CI: 0.117–0.546, *p* = 0.0001) than those without other psychiatric medications. Furthermore, patients with fluctuating plasma lithium level were less likely (20.7%) to respond to lithium (AOR = 0.207; 95% CI: 0.073–0.583, *p* = 0.003) than patients with a stable therapeutic level for most of the treatment period.

**Table 4:** Bivariate and multivariable logistic regression analysis of clinical and demographic variables for lithium response among patients with bipolar disorder at AMSH, Ethiopia (April 2018–January 2021; *n* = 262).

Outcome variables		Response (*n*, %)		Univariate analysis		Multivariable analysis	
	Category	Good responders	Nonresponders	COR (95% CI)	*p* value	AOR (95% CI)	*p* value
Age category	18–29.9	13 (18.0)	65 (34.2)	1.00		1.00	
	30–44.9	48 (66.7)	89 (46.8)	2.670 (1.35–5.4)	0.005	2.86 (1.31–6.2)	0.008 ^∗^
	≥ 45	11 (15.3)	36 (19.0)	1.53 (0.62–3.8)	0.356	1.55 (0.58–4.2)	0.385
Alcohol use	No	35 (48.6)	113 (59.5)	1.00			
	Yes	37 (51.4)	77 (40.5)	1.55 (0.90–2.7)	0.115	1.64 (0.88–3.1)	0.122
Li level in the blood	Normal range (0.6–1.2 mEq/L)	63 (75.0)	127 (71.3)	1.00		1.00	
	Fluctuating (< 0.6 or > 1.2 mEq/L)	5 (6.0)	51 (28.7)	0.20 (0.08–0.5)	0.001	0.207 (0.07–0.583)	0.003 ^∗^
Concurrent medications with Li	No	28 (38.9)	32 (16.8)	1.00		1.00	
	Yes	44 (61.1)	158 (83.2)	0.32 (0.17–0.6)	*p* < 0.001	0.253 (0.12–0.6)	*p* < 0.001^∗^
Li dose category	300 mg	3 (4.2)	5 (2.6)	1.00		1.00	
	600 mg	18 (25)	44 (23.2)	0.68 (0.15–3.2)	0.624	0.148 (0.02–1.3)	0.087
	900 mg	47 (65.3)	115 (60.5)	0.68 (0.16–2.9)	0.609	0.207 (0.03–1.7)	0.143
	1200 mg and above	4 (5.6)	26 (13.7)	0.27 (0.43–1.5)	0.133	0.125 (0.01–1.2)	0.082

*Note:* Variables included for univariate analysis: sex, age category, tobacco use, khat use, alcohol use, hospital admission, number of hospital admission, comorbidities, BMI category, is a psychiatric inpatient, presence of other psychiatric diagnosis, level of adherence, dose of Li, duration of Li therapy, plasma Li level, concurrent medications with Li, number of concurrent medications. Variables included in the multivariate analysis: age category, Li plasma level, alcohol use, concurrent medications with Li, and dose of Li. Plasma lithium levels are based on concentrations measured ≥ 5 days after any dose change with at least two measurements obtained ≥ 3 months apart, documented in the medical records over the treatment period. a). Normal lithium levels: steady‐state maintenance plasma concentrations all within the therapeutic range of 0.6–1.2 mEq/L and demonstrating low variability (coefficient of variation ≤ 20%) b). Fluctuating lithium levels: presence of one or more measurements outside the therapeutic range and/or high variability (coefficient of variation > 20%) across available steady‐state measurements.

Abbreviations: AOR, adjusted odds ratio; CI, confidence interval; COR, crude odds ratio.

^∗^ *p* values.

### 3.3. Lithium Adverse Effects

**Figure 1:**
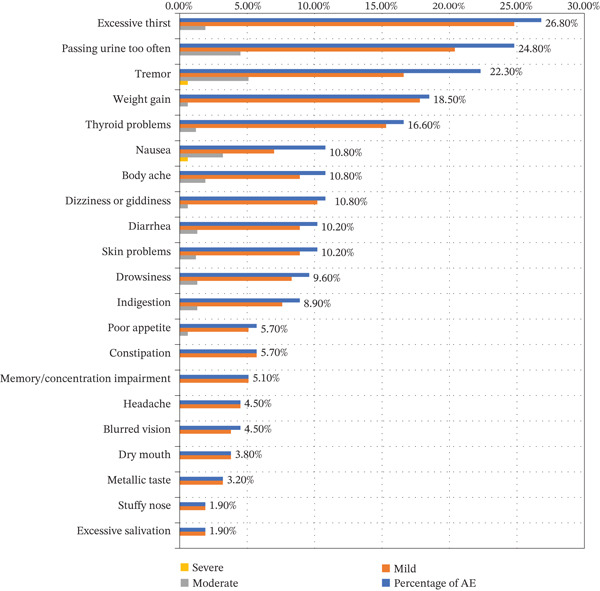
Adverse effects among patients with bipolar disorder treated with lithium at AMSH (*n* = 157).

Figure 1 shows that out of the 157 patients receiving lithium at AMSH, 92 (58.6%) of the patients had a total of 340 adverse events. The most common adverse effects were excessive thirst (42, 26.8%), frequent urination (38, 24.2%), tremor (35, 22.3%), weight gain (29, 18.5%), and thyroid issues (26, 16.6%). Less frequently, but as unpleasant, were indigestion, skin issues, constipation, diarrhea, drowsiness, body aches, and nausea. Only two cases (0.5%) of severe adverse effects were documented, whereas the majority of adverse effects (86.7%) were mild, and the remaining 11.8% were moderate in intensity.

#### 3.3.1. Comparison of Demographic and Disease Course Characteristics

Table 5 presents the baseline clinical, sociodemographic, and treatment details of individuals who experienced adverse effect and those who did not. No significant differences were observed in gender, marital status, housing type, education level, alcohol use, smoking status, BMI, comorbidities, inpatient psychiatric status, and treatment adherence between those with and without adverse effects. However, cannabis use (*p* = 0.037), hospital admissions (*p* = 0.048), lithium dose category (*p* = 0.027), and the number of other medications taken with lithium (*p* = 0.045) were significantly different between the two groups.

**Table 5:** Baseline clinical, sociodemographic, and treatment characteristics of those who experienced adverse effects and those who did not among patients with bipolar disorder at AMSH, Ethiopia (April 2018–January 2021).

Sociodemographic characteristics		Without adverse effects (*n*, %) (*n* = 65)	With adverse effects (*n*, %) (*n* = 92)	*χ* ^2^/*t*‐test	*p* value
Sex	Male	30 (46.2)	36 (39.1)	0.771	0.380
	Female	35 (53.8)	56 (60.9)		
Age	Mean (SD)	34.4 (9.8)	36.54 (9.4)		0.168
Age category	18–29.9	25 (38.5)	24 (26.1)	3.126	0.210
	30–44.9	30 (46.1)	47 (51.1)		
	≥ 45	10 (15.4)	21 (22.8)		
Marital status	Never married	38 (58.5)	49 (53.3)	4.454	0.348
	Married	19 (29.2)	22 (23.9)		
	Divorced	4 (6.2)	14 (15.2)		
	Widowed	3 (4.6)	3 (3.3)		
	Separated	1 (1.5)	4 (4.3)		
Current living arrangement	Lives alone	13 (20)	14 (15.2)	2.732	0.604
	Lives with spouse/partner	18 (27.7)	22 (23.9)		
	Lives with parents	22 (33.9)	38 (41.3)		
	Lives with other relatives	11 (16.9)	17 (18.5)		
	Others	1 (1.5)	0 (0.0)		
Level of education	No education	5 (7.7)	0 (0.0)	11.461	0.075
	Did not complete primary school	12 (18.5)	18 (19.6)		
	Completed primary school	5 (7.7)	6 (6.5)		
	Did not complete secondary school	15 (23.1)	15 (16.3)		
	Completed secondary school	7 (10.8)	21 (22.8)		
	Did not complete college education	6 (9.2)	11 (11.9)		
	Completed college education	15 (23.1)	20 (21.7)		
Tobacco use	Yes	21 (32.3)	21 (22.8)	1.748	0.186
	No	44 (67.7)	71 (77.2)		
Alcohol use	Yes	28 (43.1)	41 (44.6)	0.034	0.853
	No	37 (56.9)	51 (55.4)		
Khat use	Yes	28 (43.1)	28 (30.4)	2.653	0.103
	No	37 (56.9)	64 (69.6)		
Cannabis use	Yes	3 (4.6)	0 (0.0)	4.329	0.037 ^∗^
	No	62 (95.4)	92 (100)		
Body mass index	< 18.5	1 (1.5)	2 (2.2)	7.290	0.063
	18.5–24.9	38 (58.5)	35 (38.0)		
	25–29.9	14 (21.5)	36 (39.1)		
	≥ 30	12 (18.5)	19 (20.7)		
Hospital admission	No	58 (89.2)	70 (76.1)	3.900	0.048 ^∗^
	Yes	7 (10.8)	21 (22.8)		
Comorbid conditions	No	41 (63.1)	49 (53.3)	1.500	0.221
	Yes	24 (36.9)	43 (46.7)		
Currently admitted	No	39 (60.0)	45 (48.9)	1.959	0.162
	Yes	3 (4.6)	9 (9.8)		
Presence of other psychiatric diagnosis	No	40 (61.5)	58 (63.0)	0.113	0.737
	Yes	18 (27.7)	23 (25.0)		
No. of other psychiatric medications	Only one medication	14 (21.5)	36 (39.1)	0.150	0.928
	Two medications	51 (78.5)	35 (38.1)		
	Three medications	6 (9.2)	19 (20.7)		
Nonpsychiatric medications	No	6 (9.2)	7 (7.6)	0.132	0.716
	Yes	59 (90.8)	85 (92.4)		
Level of Li adherence	Not available	5 (7.7)	7 (7.6)	0.996	0.802
	No adherence problem	49 (75.4)	64 (69.6)		
	Intermediate adherence/some treatment interruption	10 (15.4)	18 (19.6)		
	Poor adherence/greater treatment interruption	1 (1.5)	3 (3.3)		
Li dose category	300 mg	0 (0.0)	5 (5.4)	9.164	0.027 ^∗^
	600 mg	12 (18.5)	22 (23.9)		
	900 mg	49 (75.4)	51 (55.4)		
	≥ 1200 mg	4 (6.2)	14 (15.2)		
Duration of Li therapy	Less than 1 year	9 (13.8)	21 (22.8)	2.568	0.463
	1–2 years	16 (24.6)	20 (21.7)		
	2–5 years	34 (52.3)	46 (50.0)		
	5 years and above	6 (9.2)	5 (6.5)		
Concurrent medications with Li	No	14 (21.5)	12 (13.0)	1.989	0.158
	Yes	51 (78.5)	80 (87.0)		
No. of concurrent medications with Li	No other medication	14 (21.5)	12 (13.0)	8.027	0.045 ^∗^
	One medication	51 (78.5)	71 (77.2)		
	Two medications	0 (0.0)	7 (7.6)		
	Three medications	0 (0.0)	2 (2.2)		
ALDA A	Mean (SD)	8.88 (1.0)	8.66 (1.1)		0.209
ALDA total score	Mean (SD)	5.55 (1.8)	5.21 (1.7)		0.221
Response category	No response	41 (63.1)	64 (69.6)	0.724	0.395
	Good response	24 (36.9)	28 (30.4)		

*Note:* Nonpsychiatric medications refers to the last recorded medications used during data collection time.

Abbreviation: SD, standard deviation.

^∗^ *p* < 0.05.

#### 3.3.2. Predictors for Lithium Adverse Effect

Similar to the analysis of lithium response, the univariate analysis of adverse effects considered sex, age category, tobacco use, khat use, alcohol use, hospital admission status and number of hospital admissions, presence of comorbidities, BMI category, psychiatric inpatient status, presence of other psychiatric diagnoses, level of adherence, lithium dose, duration of lithium therapy, plasma lithium level, use of concurrent medications with lithium, and number of concurrent medications. However, only age category, hospital admission, comorbidity, tobacco use, khat use, current psychiatric inpatient, concurrent use of other psychiatric medications, length of lithium therapy, and duration of lithium therapy met the inclusion criteria (*p* < 0.2) in the bivariate analysis and were included in the multivariable regression (Table 6). After multivariable analysis, age category, tobacco usage, and length of lithium medication were found to be significantly associated with adverse effects. The logistic regression model demonstrated a good fit to the data, as indicated by a nonsignificant Hosmer–Lemeshow goodness‐of‐fit test (*χ* ^2^ = 4.71, df = 8, *p* = 0.788). Patients aged 30–44.5 (AOR = 0.14, 95% CI: 0.02–0.745; *p* = 0.022) and over 45 years (AOR = 0.03, 95% CI; 0.01–0.78; *p* = 0.035) were less likely to experience adverse effects compared with those aged 18–29.9. Patients with a history of tobacco use (ever use) showed a higher odd of experiencing adverse effects compared with nonusers (AOR = 5.04, 95% CI: 1.10–23.14; *p* = 0.038). The incidence of adverse effects was also significantly associated with duration of therapy. Patients on lithium for 1–2 years (AOR = 4.85, 95% CI: 1.19–19.67, *p* = 0.027) and for more than 5 years (AOR; 10.92, 95% CI: 1.27–93.70, *p* = 0.029) were more likely to experience adverse effects compared with those on treatment for less than a year.

**Table 6:** Bivariate and multivariable logistic regression analysis of clinical and demographic variables for lithium adverse effects among patients with bipolar disorder at AMSH, Ethiopia (April 2018–January 2021; *n* = 157).

Variables	Categories	Adverse effects (*n*, %) *n* = 157		Univariate analysis		Multivariate analysis	
		Yes	No	COR (95% CI)	*p*value	AOR (95% CI	*p* value
Age category	18‐29.9	24 (15.3%)	25 (15.9%)	1.00		1.00	
	30–44.9	47 (29.9%)	30 (19.1%)	0.61 (0.30–1.3)	0.185	0.14 (0.02–0.8)	0.022 ^∗^
	≥ 45	21 (13.4%)	10 (6.4%)	0.46 (0.18–1.2)	0.102	0.03 (0.01–0.8)	0.035 ^∗^
Tobacco use	No	71 (45.2%)	44	1.00		1.00	
	Yes	21 (13.4%)	21 (13.4%)	1.61 (0.79–3.3)	0.188	5.04 (1.10–23.1)	0.038 ^∗^
Khat use	No	64 (40.8%)	37 (23.6%)	1.00		1.00	
	Yes	28 (17.8%)	28 (17.8%)	1.73 (0.89–3.4)	0.105	0.59 (0.15–2.3)	0.453
Hospital admission	No	70 (44.6%)	58 (36.9%)	1.00		1.00	
	Yes	21 (13.4%)	7 (4.5%)	0.40 (0.16–1.0)	0.053	0.53 (0.12–2.4)	0.453
Comorbidities	No	49 (31.2%)	41 (26.1%)	1.00		1.00	
	Yes	43 (27.4%)	24 (15.3%)	0.67 (0.35–1.3)	0.222	0.81 (0.28–2.4)	0.704
Currently admitted	No	45 (28.7%)	9 (5.7%)	1.00		1.00	
	Yes	39 (24.8%)	3 (1.9%)	0.38 (0.10–1.5)	0.173	0.23 (0.04–1.2)	0.083
Duration of Li treatment	Less than 1 year	21 (13.4%)	9 (5.7%)	1.00		1.00	
	1–2 years	20 (12.7%)	16 (10.2%)	1.87 (0.67–5.2)	0.231	4.85 (1.19–19.7)	0.027 ^∗^
	2–5 years	46 (29.3%)	34 (21.7%)	1.72 (0.70–4.2)	0.234	2.42 (0.66–8.9)	0.182
	Above 5 years	5 (3.2%)	6 (3.8%)	2.8 (0.68–11.6)	0.155	10.92 (1.27–93.7)	0.029 ^∗^
Concurrent medications with Li	No	12 (7.6%)	14 (8.9%)	1.00		1.00	
	Yes	80 (50.9%)	51 (32.5%)	0.55 (0.23–1.3)	0.162	0.98 (0.26–3.7)	0.979

*Note:* Variables included for univariate analysis: sex, age category, tobacco use, khat use, alcohol use, hospital admission, number of hospital admission, comorbidities, BMI category, currently admitted, presence of other psychiatric diagnosis, level of adherence, dose of Li, duration of Li therapy, plasma Li level, concurrent medications with Li, and number of concurrent medications. Variables included in the multivariate analysis: age category, tobacco use, khat use, hospital admission, comorbidities, currently admitted, duration of Li therapy, and concurrent medications with Li.

Abbreviations: AOR, adjusted odds ratio; CI, confidence interval; COR, crude odds ratio.

^∗^ Significant association (*p* < 0.05).

## 4. Discussion

A variety of medications are available to manage the symptoms of BD, but individual responses to treatment can vary widely due to the complexity of the condition and other factors. Lithium, a mood stabilizer, is considered the first‐line treatment for BD [10, 26]. Although some patients respond well to lithium, tolerate it effectively, and remain stable for years, others do not have the same experience. Because there are no reliable predictors of response, determining the best treatment plan often involves months of trial and error, which only adds suffering to the patient [12]. This study′s primary goal was to look into the clinical response and adverse effects of lithium use in clinically representative samples of patients with BD at AMSH. This study included 262 well‐characterized cases of patients with BD receiving lithium treatment for at least 6 months. In this retrospective analysis, 27.5% of the patients were identified as GRs, with a total Alda score of 7 or higher. Several studies have used the Alda scale to evaluate the response to mood stabilizers, though the response criteria have varied. In an earlier report by Garnham et al. (2007) [27], individuals with a total score of 7 or higher were classified as “full responders,” with a response rate of 30%. Similarly, the lithium genetics research consortium [20] applied the same criteria and found that 33% of lithium‐treated patients were categorized as full responders. In the current study, the mean total score (3.52 ± 0.085) was lower compared to the consortium′s findings, where the initial clinical report showed a mean score of 4.4 ± 3.1 [20], and to a subsequent genome‐wide association analyses that reported scores of 4.3 ± 3.3 and 3.9 ± 3.0 [28]. However, the mean A score of the present study (8.7 ± 0.066) was higher than that of the ConLiGen study, whose scores ranged from 6.0 to 6.4 [20, 28]. Although the mean A score was higher in the present study, suggesting a reduction in bipolar symptoms, this was accompanied by a higher B score. The B score considers baseline clinical factors that could influence the actual causal relationship between treatment and outcome, including prior mood episodes, treatment duration, adherence, and the use of other medications. The net effect was a lower total Alda score, probably attributed to a prolonged lithium treatment and concomitant therapy with other psychotropic medications, which were largely observed in the IRs.

Direct comparisons of the response between the present study and previous research are challenging as earlier studies often focused on a single mood stabilizer, while the current study considered responses from multiple medications. To examine long‐term responses in a naturalistic clinical setting, it is essential to consider changes in medication and combinations of different mood stabilizers over the course of treatment. However, the findings of the current study are comparable to other studies. In a study conducted in Korea by Ahn et al. (2017) [29], 34% of patients (*n* = 80) with BD responded well to mood stabilizer medications. Similarly, a different trial with 78 participants found a 30% full response to lithium [30]. In contrast, a study by Sportiche et al. (2017) [31] in French university‐affiliated facilities showed that only 17% of patients (*n* = 300) with BD were classified as GRs, which is lower than the response rate observed in the present study.

The relationship between various factors and lithium responsiveness in BD has been extensively studied. Different predictors of lithium response have been identified in earlier studies, such as age at onset, family history, history of episodes with mixed features, number of prior episodes, alcohol consumption, rapid cycling, psychotic characteristics, and familial history of BD [22, 30–32]. In the present study alcohol use and concurrent use of other drugs were significantly associated with lithium response in the univariate analysis, but this association did not persist in the multivariable logistic regression analysis. Age, variations in plasma level of lithium, and concurrent use of psychiatric medications were significantly associated with lithium response. A similar study by Laroche et al. [33] also indicates that plasma lithium levels are significant predictors of treatment response. Fluctuations in lithium plasma levels correlate with a 15.5% response rate, significantly lower than those maintaining stable therapeutic levels (*p* = 0.003). Although these findings highlight the importance of plasma stability, other factors like age and concurrent use of psychiatric medications also play roles in treatment efficacy, suggesting that a multifaceted approach to understanding lithium responsiveness is necessary [34]. Variations in lithium level may reflect adherence, and inadequate adherence has been proposed as one potential mechanism underlying the so‐called “lithium resistance” [35].

Although lithium is effective in managing mood disorders, adverse effects often lead patients to discontinue its use [36]. No studies have yet examined the occurrence of lithium‐related adverse effects in Ethiopian populations. According to the current study, more than half of the participants (58.6%) reported experiencing at least one adverse effect. Studies have shown that 75 to 90% of patients on long‐term lithium therapy experience one or more adverse effects [37]. Nephrogenic symptoms were the most commonly reported, with 26.8% of patients experiencing polydipsia, 24.2% reporting polyuria, and 3.8% experiencing dry mouth. These findings are consistent with other studies [38] that reported increased thirst and urination in nearly 70% of the study participants. According to a meta‐analysis [39], lithium reduces urine‐concentrating ability to 15% of its normal maximum and decreases glomerular filtration rates by 6.22 mL/min. Although the absolute risk of renal failure was only 0.5% in the meta‐analysis [39], it remains a serious concern. Therefore, serum creatinine levels should be monitored regularly, and the decision to continue/discontinue lithium treatment should be individualized through consistent follow‐up and careful balancing of the benefits and the risks to avoid irreversible kidney damage.

The current study has also found that 22.3% of patients on lithium therapy reported experiencing tremors. Studies have documented a wide range of tremor occurrences, from 4% to 65%, with an average frequency of about 27% among lithium‐treated patients. Hand tremors are a well‐recognized adverse effect of lithium, but accurately determining their onset and severity remains a challenge for healthcare providers [40, 41]. Along with tremors, gastrointestinal issues such as nausea (10.8%), diarrhea (10.2%), and indigestion (8.9%) are frequently reported by patients, with earlier studies showing that up to half of those on lithium may experience one or more of these symptoms [42].

Weight gain is a common and significant concern for individuals with BD. In this study, 18.5% of patients on lithium therapy reported weight gain, which is consistent with earlier research showing that 25%–50% of patients experience this adverse effect [43, 44]. A 7‐year prospective study indicated that weight gain typically occurs during the first 2 years of lithium treatment before stabilizing [45]. Potential underlying causes may include factors like fluid retention, increased appetite, lithium‐induced subclinical hypothyroidism, or a mix of biological and genetic factors [46].

The present study also found that 16.6% of the patients experienced thyroid‐related problems. Among patients treated with lithium, the reported rates of overt and subclinical hypothyroidism range from 8% to 19% and 23%, respectively [47]. In the Chinese population, the incidence of hypothyroidism among lithium‐treated patients was higher (28%–32%) than in those not receiving lithium treatment (6.3%–10.8%) [48]. A meta‐analysis showed that hypothyroidism was more common in lithium‐treated patients than in controls, with an average increase in TSH levels of 4.00 iU/mL [39]. The prevalence of goiter is estimated at 30%–59% of lithium‐treated patients. Lithium‐induced goiter may result from inhibited thyroid hormone release, raising TSH and increasing thyroid volume, along with lithium′s activation of tyrosine kinase and effects on cyclic AMP and Wnt/beta‐catenin pathways, which stimulate thyrocyte proliferation [49]. It is important to consider that the diagnoses of metabolic abnormalities in this study were based on available medical records, which may have underestimated the actual presence of such conditions.

Additionally, cognitive effects were reported by 5% of the study participants including memory and concentration impairment. Cognitive problems are a common challenge for patients with BD, and studies have demonstrated impaired cognitive functioning across various domains, regardless of treatment [50]. Dermatological adverse effects from lithium treatment, such as acne and psoriasis, can be distressing and may hinder treatment adherence, affecting 3.4%–45% of patients in clinical trials [51]. In this study, 10% of participants reported dermatological adverse effects, primarily acne and dry skin. Additionally, headaches and body aches were reported by 4.5% and 10.8% of patients, respectively, aligning with findings from other studies [52]. Dysgeusia, or the experience of a metallic taste, was reported by 3.2% of patients, potentially resulting from decreased salivary flow that concentrates electrolytes in the saliva, leading to this metallic sensation.

The results of this study highlight several key factors associated with adverse effects of lithium therapy, including age, tobacco use, and duration of lithium treatment. The analysis shows that patients aged 30–44.5 and over 45 were less likely to experience adverse effects from lithium compared to those aged 18–29.9. This contrasts with earlier research that suggests an increase in adverse effects with age [53]. In the present study, age was not significantly correlated with treatment duration (Pearson *r* = 0.069, *p* = 0.263), indicating that older age does not necessarily imply longer exposure. One possible explanation is survivor bias: Older patients who experienced severe adverse effects early may have discontinued lithium, leaving those who tolerate treatment well. Additionally, older patients might experience early gastrointestinal adverse effect such as diarrhea and nausea but continue therapy due to perceived benefits, whereas younger patients might be more sensitive to lithium due to a faster metabolism or higher initial serum levels. Some studies also suggest that younger patients are often prescribed higher starting doses, leading to increased reports of nausea, tremors, and cognitive blunting during early treatment [54]. Similarly, adolescents have been found to report a higher frequency of these adverse effects during lithium therapy [55]. These age‐related differences emphasize the importance of age‐specific monitoring when managing lithium in patients with BD.

In the present study, tobacco use was associated with a higher likelihood of lithium‐related adverse effects after adjustment for relevant covariates. Although the bivariate analysis showed a higher but nonsignificant odd of adverse effects among tobacco users, the association became stronger and statistically significant in the multivariable model, suggesting the presence of negative confounding in the unadjusted analysis. This indicates that the independent association of tobacco use was masked by other factors unevenly distributed between smokers and nonsmokers. This association supports existing data that suggest that tobacco use can affect drug metabolism, potentially worsening adverse effects by altering lithium′s and other concomitant medications′ processing in the body [56]. Another possibility is that smoking exacerbates psychiatric symptoms, such as anxiety and depression, which could indirectly affect patients′ experience of adverse effects. Smokers with BD may also experience greater mood instability due to nicotine′s impact on the brain′s dopamine system, potentially influencing their tolerance to lithium [57]. Other nonpharmacokinetic factors like behavioral difference in symptom reporting, lifestyle factors, and unmeasured comorbidities may further contribute to the higher likelihood of reported adverse effects among tobacco users.

Duration of lithium therapy was also significantly associated with adverse effects. Patients on lithium for 1–2 years had nearly five times higher odds of experiencing adverse effects, whereas those on lithium for more than 5 years had more than 10 times higher odds compared with patients treated for less than a year. This association may be attributed to the cumulative effect of lithium over time, where prolonged exposure to the drug increases the likelihood of adverse effects like tremors, thyroid dysfunction, and renal impairment as described elsewhere [58]. This supports previous research suggesting that long‐term lithium use requires careful management to balance the therapeutic benefits with potential adverse effects. Although the association of age and treatment duration may appear contradictory, risk increased in longer duration but was lower in older patients, these observations are independent. In the present study, older age did not necessarily correspond with longer treatment duration, nor does younger age imply a shorter duration. As shown in Table 6, only a small number of patients had taken lithium for more than 5 years, which limits the influence of this category on the age distribution, suggesting that the observed relationship between age and adverse effects is independent of treatment duration.

### 4.1. Limitations of the Study

Despite the use of a standardized tool to measure lithium response in a real‐world clinical setting, several limitations should be acknowledged. The retrospective design relies on routinely recorded clinical data which may be incomplete or inconsistently documented, potentially influencing B‐scale scoring and treatment categorization. Alda scale ratings were conducted by a single trained rater without formal interrater reliability testing, which may affect reproducibility. Adverse effect data were not available for all participants, introducing potential selection bias, and participant reported adverse effects are subject to recall bias. In addition, the sample size limited subgroup analyses. Finally, although the study setting serves a broad referral population, its specialized nature may limit generalizability to primary care or less severe clinical settings.

## 5. Conclusions

In conclusion, out of 262 patients with BD receiving lithium treatment, only 27.5% of participants were categorized as GRs, underscoring the difficulties in achieving optimal treatment outcomes. The study identified that age, concurrent use of psychiatric medications, and plasma lithium levels were significantly associated with treatment response. Additionally, most patients reported experiencing at least one adverse event, with tremor, excessive thirst, and frequent urination being the most common. Although the majority of adverse effects were classified as mild to moderate, increased incidence of adverse effects was associated with tobacco use and younger age. This suggests that demographic and lifestyle factors may significantly impact the safety profile of lithium therapy. These results highlight the necessity for personalized treatment approaches in managing BD, taking into consideration individual patient characteristics to improve both therapeutic efficacy and safety. Future research should focus on elucidating the mechanisms that underlie these associations to further improve lithium treatment strategies.

**Nomenclature**
AMSH: Amanuel Mental Specialized Hospital
BD: bipolar disorder
NeuroGAP: Neuropsychiatric Genetics of African Populations

## Author Contributions

All authors have contributed equally to this paper starting form study design, data collection, analysis and interpretation, drafting of the manuscript, and critical revision of the manuscript for important intellectual content. The guarantor is Ephrem Engidawork.

## Funding

This study received generous funding and data support from the Neuropsychiatric Genetics of African Populations‐Psychosis, Ethiopia (NeuroGAP‐P‐E) project (NIH Grant No. R01MH120642). Additional support was provided by Addis Ababa University as part of a PhD dissertation.

## Disclosure

The funders did not have any role in the design, analysis, writing, and publication of the work.

## Ethics Statement

This study is part of the NeuroGAP project, which received ethical approval from the Institutional Review Board of the College of Health Sciences, Addis Ababa University (protocol 014/17/Psy) and also obtained specific approval for this subproject from the same IRB (protocol 085/18/Pharmacy). Permission to conduct the study was also obtained from ASMH. Furthermore, for the interview component, the subjects or their legal guardians provided written informed consent after being informed about the study′s objectives, procedures, anticipated results, potential hazards, and associated discomforts. For the retrospective chart review, data were extracted from medical records, and patient informed consent was waived. All methods comply with relevant guidelines and regulations.

## Conflicts of Interest

The authors declare no conflicts of interest.

## Data Availability

Data are available upon reasonable request. The data are available in the public library of Addis Ababa University.
